# Prevalence, associated factors, and prognostic value of P wave abnormality in patients with coronary artery disease

**DOI:** 10.1016/j.ijcrp.2025.200533

**Published:** 2025-10-24

**Authors:** Kazutoshi Hirose, Hiroyuki Kiriyama, Shun Minatsuki, Yugo Nagae, Tatsuki Furusawa, Takashi Hiruma, Atsushi Kobayashi, Masataka Sato, Shinnosuke Sawano, Tatsuya Kamon, Hiroki Shinohara, Akihito Saito, Satoshi Kodera, Junichi Ishida, Hiroyuki Morita, Norihiko Takeda

**Affiliations:** a Department of Cardiovascular Medicine, The University of Tokyo, Tokyo, Japan; b Department of Healthcare Information Management, The University of Tokyo, Tokyo, Japan

**Keywords:** P-wave terminal force in V1, Cardiovascular outcome, Coronary artery disease, Percutaneous coronary intervention, Prognostic value

## Abstract

**Background:**

P-wave terminal force in V1 (PTFV1) on electrocardiography is an easily available and cost-effective surrogate marker reflecting myocardial electrical and structural remodeling. An abnormal PTFV1 was recently suggested to be a reliable predictor of adverse cardiovascular events, whereas its performance in the setting of coronary artery disease (CAD) remains unknown.

**Methods:**

We retrospectively investigated 3147 patients with CAD who underwent percutaneous coronary intervention at our institution. Abnormal PTFV1 was defined as PTFV1 >0.04 mm⋅s. The primary outcome was a composite of cardiac death and heart failure hospitalization. The Cox proportional hazard models were constructed to investigate the association between PTFV1 and clinical outcome.

**Results:**

Among the study population, 592 (18.8 %) patients had abnormal PTFV1. Patients with abnormal PTFV1 had worse atherosclerotic and cardiovascular profiles, higher concentrations of brain natriuretic peptide and C-reactive protein, and more advanced CAD than those with normal PTFV1 (p < 0.05). The abnormal PTFV1 group had more pronounced left ventricular and atrial remodeling than normal PTFV1 group (p < 0.05), but the association between left atrial size and PTFV1 was not significant after multivariable adjustment (p = 0.355). During a median follow-up of 5.2 years, patients with abnormal PTFV1 more frequently experienced the primary outcome than those with normal PTFV1 (log-rank p < 0.001). Abnormal PTFV1 carried a significant risk for the primary outcome independent of baseline characteristics, biomarkers, and angiographic features (adjusted hazard ratio 2.38, p < 0.001).

**Conclusions:**

In patients with CAD who undergo percutaneous coronary intervention, abnormal PTFV1 is a robust and independent risk factor for adverse cardiovascular outcomes.

## Highlights

• Among patients with coronary artery disease (CAD), 18.8 % had abnormal P-wave terminal force in V1 (PTFV1).
• Patients with abnormal PTFV1 were accompanied by more advanced myocardial remodeling and a greater burden of CAD.
• Abnormal PTFV1 carried an independent risk for adverse outcomes including cardiac death and heart failure hospitalization.
• The prognostic value of PTFV1 could enhance early, accessible, and economical risk stratification in patients with CAD.

## Graphical abstract

**Figure:**
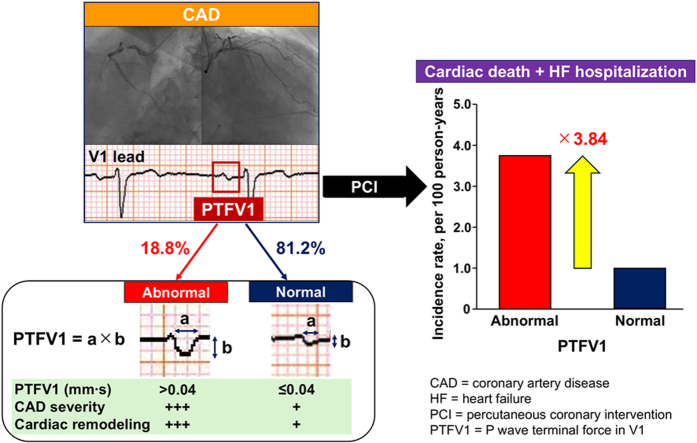

## 1 Introduction

Coronary artery disease (CAD) is a major healthcare burden, affecting 240 million people worldwide [1]. Despite advances in therapeutic strategies, such as medical treatment and revascularization [2,3], patients with CAD still have an elevated risk of incident heart failure (HF) and mortality [1,[4], [5], [6], [7]]. This situation highlights the requirement for early identification of patients who are susceptible to adverse cardiovascular events.

P-wave morphological changes on electrocardiography (ECG) are easily available and cost-effective surrogate markers of myocardial electrical and structural remodeling [[8], [9], [10], [11]]. These changes could be related to poorer clinical outcomes in some clinical settings [12,13]. P-wave terminal force in V1 (PTFV1), which is a negative component of the P wave in lead V1, has emerged as a reliable predictor of adverse cardiovascular events, such as incident atrial fibrillation (AF) and sudden cardiac death in the general population [12,14,15], ischemic stroke in patients with or without AF [16,17], and HF-related events in patients with chronic HF [18]. However, the performance of PTFV1 in the setting of CAD remains unclear. Determining the role of PTFV1 in predicting adverse cardiovascular outcomes could aid in early risk stratification and enhance preventive strategies in patients with CAD. Therefore, this study aimed to investigate the prevalence of abnormal PTFV1, the association between PTFV1 and myocardial remodeling, and the prognostic value of PTFV1 for long-term cardiovascular events in patients with comprehensive CAD, including acute coronary syndrome (ACS) and chronic coronary syndrome (CCS).

The novel contributions of the present study were to clarify (i) the burden of abnormal PTFV1, (ii) its association with left atrial (LA) structural remodeling, and (iii) its independent and incremental prognostic value for adverse cardiovascular outcomes in patients with CAD.

## 2 Methods

### 2.1 Study population

We retrospectively enrolled 3821 consecutive patients with CAD who underwent percutaneous coronary intervention (PCI) at The University of Tokyo Hospital between January 2006 and December 2020. Patients with a history of AF (n = 343) or implantation of a cardiac electronic device (n = 46), non-sinus rhythm on ECG (n = 107), or a lack of ECG data (n = 178) at the time of index PCI were excluded, based on the analyzability of P-wave indices and the potential impact on P-wave morphology and its association with CAD. Therefore, the final study population comprised 3147 patients. The requirement for written informed consent was waived because of the retrospective nature of the study. The study was conducted in accordance with the principles of the Declaration of Helsinki and approved by the Institutional Review Board of The University of Tokyo (2021238NI-(2)).

### 2.2 Clinical data collection

The patients’ demographics, medical history, laboratory examination findings, echocardiographic parameters, and angiographic findings at the initial hospitalization were obtained from a review of electronic medical records. The body mass index was calculated using height and weight (kg/m^2^) at the time of admission. Laboratory parameters, including renal function, lipid and glycemic profiles, and concentrations of C-reactive protein (CRP) and brain natriuretic peptide (BNP), were obtained within 3 months before and at the admission. The estimated glomerular filtration rate was computed by the Modification of Diet in Renal Disease equation [19], and chronic kidney disease (CKD) was determined by an estimated glomerular filtration rate <60 mL/min/1.73 m^2^ for >3 months [1].

**Fig. 1:**
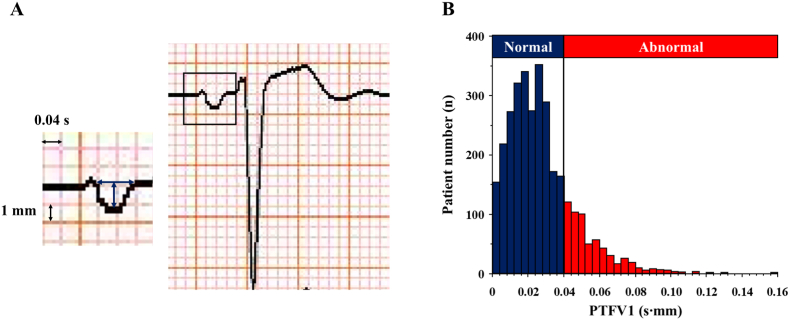
(A) Representative electrocardiogram for determining PTFV1. PTFV1 (mm∙s) was calculated by the duration (s) of the terminal negative part of the P wave in lead V1 multiplied by the absolute value of its amplitude (mm). (B) Distribution of PTFV1 in patients with CAD who underwent PCI. The red bars indicate abnormal PTFV1, and the blue bars show normal PTFV1. CAD = coronary artery disease, PCI = percutaneous coronary intervention, and PTFV1 = P-wave terminal force in V1. (For interpretation of the references to colour in this figure legend, the reader is referred to the Web version of this article.)

Resting 12-lead ECGs within 3 months before and 1 month after the admission were extracted to assess P-wave morphology in all patients. A conventional 12-lead ECG was recorded in 10 mm/mV and 25 mm/s utilizing an automated ECG machine (FCP-7541 and FCP-8700, Fukuda Denshi Co., Ltd., Tokyo, Japan). Analog-to-digital conversion was performed by Fukuda signal acquisition module at a sampling rate of 500 Hz. The P-wave amplitude and the duration in lead V1 were measured by an experienced cardiologist who was blinded to the patients’ clinical information. These parameters were also automatically analyzed by the MBF-1000 ECG Data Management System (Fukuda Denshi Co., Ltd., Tokyo, Japan) in 1179 patients. PTFV1 (mm⋅s) was calculated as the duration (s) of the terminal negative part of the P wave in lead V1 multiplied by the absolute value of its amplitude (mm) (Fig. 1A) [13,18,20,21]. Abnormal PTFV1 was defined as PTFV1 >0.04 mm⋅s, which was based on the cut-point related to adverse cardiovascular outcomes in previous studies [12,15,17,21]. An excellent correlation was observed between automatically and manually measured PTFV1 in 20 randomly selected patients (r = 0.95), and its mean variability was 0.001 ± 0.012 mm⋅s [mean ± 1.96 standard deviation (SD)] in a Bland–Altman analysis. Intra-observer variability of PTFV1 was also adequate, with an excellent correlation (r = 0.97) and a low mean difference of 0.0002 ± 0.006 (mean ± 1.96 SD).

Echocardiographic measurements within 1 year before and 3 months after the index admission were also collected in 1470 patients. The dimensions of the cardiac chambers were measured in the standard manner [22]. The left ventricular (LV) ejection fraction was assessed by the Teichholz or biplane Simpson method. The LV mass index was calculated using the following formula: LV mass = 0.8 × 1.04 × [(LV end-diastolic dimension + posterior wall thickness + interventricular septum thickness)^3^−(LV end-diastolic dimension)^3^]+0.6, which was indexed to body surface area [22]. The LA anteroposterior diameter was acquired in the parasternal long-axis view. Transmitral inflow signals were evaluated to derive peak early (E) and late diastolic velocity [23]. Peak early diastolic velocity (e’) of the septal and lateral mitral annulus was obtained from tissue Doppler imaging and averaged, and E/e’ ratio was then calculated [23].

Angiographic data on the numbers and types of diseased vessels were also acquired from the patients’ medical records. A visually estimated diameter stenosis ≥70 % for non-left main trunk (LMT) disease and ≥50 % for LMT disease was defined as clinically significant stenosis [24]. A LMT artery lesion was considered as a two-vessel disease comprising the left anterior descending and left circumflex arteries. PCI was carried out in accordance with established guidelines [24].

### 2.3 Study outcomes

The primary outcome was a composite of cardiac death and HF hospitalization. Cardiac death comprised death resulting from ischemic heart disease, fatal arrhythmia, HF, cardiogenic shock, or sudden death. Incident HF was determined by the Framingham criteria [25]. The secondary outcomes were occurrence of cardiac death or HF hospitalization. These clinical events were derived from a review of electronic medical records. The study end date was December 31, 2021.

### 2.4 Statistical analysis

The patients were classified into the abnormal and normal PTFV1 groups. Continuous variables are expressed as the mean ± SD or median (interquartile range), and were compared using the unpaired Student *t*-test or the Wilcoxon rank-sum test as appropriate. Categorical variables are described as numbers and proportions, and were compared with the chi-square test. The associations between PTFV1 and the clinically relevant risk factors, laboratory parameters, angiographic features, and cardiac structural remodeling were assessed using univariable analyses in patients who received echocardiographic measurements (n = 1470), and factors with p < 0.05 were introduced into a multivariable analysis. Kaplan–Meier survival curves were used to display event-free survival and were analyzed with the log-rank test. Univariate and multivariate Cox regression models were constructed to investigate the associations between abnormal PTFV1 (>0.04 mm⋅s) and the primary or secondary outcomes. These models were adjusted for clinically relevant covariates as follows: model 1 was adjusted for age, sex, body mass index, current smoking, hypertension, diabetes mellitus, dyslipidemia and CKD; model 2 was adjusted for model 1 plus ACS, prior myocardial infarction, a history of PCI and coronary artery bypass grafting (CABG), and a history of HF hospitalization and stroke/transient ischemic attack; and model 3 was adjusted for the variables in model 2 plus biomarkers (log BNP and log CRP) and angiographic characteristics (multivessel disease, LMT involvement, and chronic total occlusion). Adjusted hazard ratios (HRs) with their 95 % confidence intervals (CIs) were reported. To determine the effect of LV and LA morphology on the relationship between PTFV1 and clinical events, we performed sensitivity Cox regression analyses in patients who had echocardiographic assessment (n = 1470). Given that the development of AF could be a potential confounding factor in the association between PTFV1 and adverse outcomes [26], sensitivity Cox regression models were constructed in patients who remained free of AF before the occurrence of the primary outcome. Subgroup analyses were also conducted between PTFV1 and the primary endpoint accounting for interaction terms. In addition, we used net reclassification analysis to evaluate the incremental prognostic value of PTFV1 over the clinically relevant variables which were introduced into the Cox regression models. A value of p < 0.05 was considered statistically significant. Statistical analyses were performed with JMP Pro 17 software (SAS Institute, Cary, NC, USA) and R v.4.4.2 (R Foundation for Statistical Computing, Vienna, Austria).

## 3 Results

### 3.1 Baseline characteristics

Among 3147 patients, the mean age was 68 ± 10 years, and 2443 (77.6 %) patients were men. The frequency of ACS was 27.4 %. Fig. 1B shows the distribution of PTFV1, and 592 (18.8 %) patients had abnormal PTFV1. The baseline characteristics according to the status of PTFV1 are shown in Table 1. Patients in the abnormal PTFV1 group had a higher prevalence of hypertension, CKD, prior myocardial infarction, the history of CABG and HF hospitalization, and more frequently received prescriptions of β-blockers and diuretics than those in the normal PTFV1 group (all p < 0.05). BNP and CRP concentrations were significantly higher in the abnormal PTFV1 group than in the normal PTFV1 group (both p < 0.001). Regarding angiographic findings, patients with abnormal PTFV1 showed a greater burden of CAD, including multivessel disease, involvement of the LMT artery and chronic total occlusion (all p < 0.01).

**Table 1:** Baseline characteristics according to the status of PTFV1.

	Abnormal PTFV1 (N = 592)	Normal PTFV1 (N = 2555)	p value
PTFV1, mm⋅s	0.050 (0.045–0.061)	0.019 (0.011–0.027)	N/A
Age, years	69 ± 10	68 ± 10	0.053
Men, n (%)	466 (78.7)	1977 (77.4)	0.481
Body mass index, kg/m^2^	24.3 ± 3.9	24.5 ± 3.9	0.557
Atherosclerotic risk factors			
Current smoking, n (%)	388 (65.5)	1660 (65.0)	0.793
Hypertension, n (%)	522 (88.2)	2159 (84.5)	0.023
Diabetes mellitus, n (%)	310 (52.4)	1326 (51.9)	0.838
Dyslipidemia, n (%)	465 (78.6)	2139 (83.7)	0.003
CKD, n (%)	277 (46.8)	936 (36.6)	<0.001
Cardiovascular comorbidities			
ACS, n (%)	156 (26.4)	706 (27.6)	0.529
Prior myocardial infarction, n (%)	75 (12.7)	249 (9.8)	0.035
History of PCI, n (%)	91 (15.4)	389 (15.2)	0.929
History of CABG, n (%)	79 (13.3)	138 (5.4)	<0.001
HF hospitalization, n (%)	37 (6.3)	45 (1.8)	<0.001
Stroke/TIA, n (%)	60 (10.1)	246 (9.6)	0.708
Medications			
β blockers, n (%)	202 (34.1)	618 (24.2)	<0.001
RAAS blockers, n (%)	324 (54.7)	1338 (52.4)	0.300
Calcium channel blockers, n (%)	284 (48.0)	1234 (48.3)	0.887
Nitrates, n (%)	118 (19.9)	481 (18.8)	0.537
Diuretics, n (%)	126 (21.3)	297 (11.6)	<0.001
Statins, n (%)	321 (54.2)	1364 (53.4)	0.713
Oral anti-diabetic drugs, n (%)	172 (29.1)	763 (29.9)	0.698
Insulin, n (%)	63 (10.6)	258 (10.1)	0.694
Laboratory data			
eGFR, ml/min/1.73m^2^ (n = 3146)	60.8 (44.7–74.4)	65.9 (53.2–78.6)	<0.001
LDL cholesterol, mg/dl (n = 3055)	96 (78–119)	99 (80–122)	0.225
HDL cholesterol, mg/dl (n = 2894)	49 (42–59)	49 (41–59)	0.482
Triglyceride, mg/dl (n = 3062)	115 (81–161)	118 (85–173)	0.066
HbA1c, % (n = 3082)	6.1 (5.7–6.9)	6.1 (5.7–6.9)	0.791
BNP, pg/ml (n = 3021)	89 (36–255)	37 (17–93)	<0.001
CRP, mg/dl (n = 3136)	0.14 (0.06–0.44)	0.10 (0.04–0.28)	<0.001
Angiographic findings			
LMT lesion, n (%)	83 (14.0)	221 (8.7)	<0.001
Multivessel disease, n (%)	464 (78.4)	1861 (72.8)	0.006
Chronic total occlusion, n (%)	149 (25.2)	454 (17.8)	<0.001

ACS = acute coronary syndrome, BNP = brain natriuretic peptide, CABG = coronary artery bypass grafting, CKD = chronic kidney disease, CRP = C-reactive protein, eGFR = estimated glomerular filtration rate, HDL = high density lipoprotein, HF = heart failure, LDL = low density lipoprotein, LMT = left main trunk artery, PCI = percutaneous coronary intervention, PTFV1 = P-wave terminal force in V1, RAAS = renin-angiotensin-aldosterone system and TIA = transient ischemic attack.

### 3.2 Association between P-wave morphology and myocardial remodeling

The relationships between PTFV1 and myocardial structural/functional parameters were assessed in 1470 patients who received echocardiographic measurements. The abnormal PTFV1 group had more advanced LV remodeling, including a larger LV size and LV mass index, a lower LV ejection fraction, and a greater E/e’ ratio, as well as more extensive LA structural remodeling than the normal PTFV1 group (all p < 0.01; Table S1). Univariable analyses showed that LV ejection fraction, LV mass index, LA diameter, and E/e’ ratio were associated with PTFV1 (p < 0.001; Table S2). However, these associations were only significant for LV mass index and E/e’ ratio (both p < 0.01), but not for LA size (p = 0.355) in the multivariable analysis (Table S2).

### 3.3 Abnormal PTFV1 and clinical outcomes

**Fig. 2:**
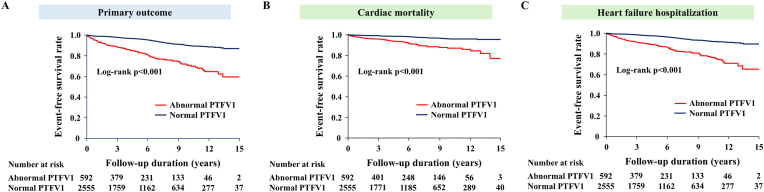
Kaplan–Meier survival curves of the composite primary outcome (A), cardiac mortality (B) and heart failure hospitalization (C) according to the status of PTFV1. PTFV1 = P-wave terminal force in V1.

During the follow-up of 5.2 (2.1–8.9) years, 265 patients experienced the primary outcome (1.45 per 100 person-years). The primary outcome occurred in 119 patients with abnormal PTFV1 (3.72 per 100 person-years) and 146 patients with normal PTFV1 (0.97 per 100 person-years). Kaplan–Meier survival curves showed poorer event-free survival in the abnormal PTFV1 group than in the normal PTFV1 group (log-rank p < 0.001; Fig. 2A). The univariate Cox proportional hazard model showed a significant association between abnormal PTFV1 and the primary outcome (p < 0.001; Table 2). In the multivariate Cox model adjusted for age, sex, and atherosclerotic risk factors, abnormal PTFV1 was independently associated with the primary outcome (model 1; Table 2), which remained significant after adjustment for cardiovascular comorbidities (model 2; Table 2). Furthermore, even after controlling for BNP, CRP, and the severity of CAD, abnormal PTFV1 was still related to the primary endpoint (adjusted HR 2.38, 95 % CI 1.83–3.09, p < 0.001; model 3; Table 2). The C-index of the final Cox model was 0.828 (95 % CI 0.802–0.855, p < 0.001), indicating a good model fit. To confirm the robustness of the relationship between PTFV1 and adverse cardiac events, we constructed univariate and multivariate Cox regression models with PTFV1 as a continuous variable (per 1-SD increase). These models showed consistent results derived from the categorical models (adjusted HR 1.26, 95 % CI 1.14–1.38, p < 0.001, model 3; Table 2), with the C-index of 0.821 (95 % CI 0.795–0.848, p < 0.001). Exploratory analysis for the primary outcome replacing cardiac mortality as all-cause death exhibited consistent results in the Kaplan-Meier curves (log-rank p < 0.001; Fig. S1) and in the multivariate models (Table S3).

**Table 2:** Cox regression models in the association of PTFV1 with the primary outcome.

	Categorical model (PTFV1 >0.04 mm⋅s)		Continuous model (per 1-SD increase)	
	HR (95 % CI)	p value	HR (95 % CI)	p value
Univariate model	3.84 (3.02–4.90)	<0.001	1.48 (1.38–1.58)	<0.001
Multivariate model 1	3.53 (2.76–4.50)	<0.001	1.51 (1.39–1.64)	<0.001
Multivariate model 2	3.21 (2.51–4.12)	<0.001	1.47 (1.34–1.60)	<0.001
Multivariate model 3	2.38 (1.83–3.09)	<0.001	1.26 (1.14–1.38)	<0.001

Multivariate model 1 = adjusted for age, sex, body mass index, current smoking, hypertension, diabetes mellitus, dyslipidemia and CKD, model 2 = adjusted for model 1 plus ACS, prior myocardial infarction, history of PCI, CABG and HF hospitalization, and stroke/transient ischemic attack, model 3 = adjusted for model 2 plus log BNP, log CRP, LMT lesion, multivessel disease and chronic total occlusion. ACS = acute coronary syndrome, BNP = brain natriuretic peptide, CABG = coronary artery bypass grafting, CI = confidence interval, CKD = chronic kidney disease, CRP = C-reactive protein, HF = heart failure, HR = hazard ratio, LMT = left main trunk artery, PCI = percutaneous coronary intervention, PTFV1 = P-wave terminal force in V1 and SD = standard deviation.

Regarding the secondary outcomes, the frequency of cardiac mortality and HF hospitalization was higher in patients with abnormal PTFV1 than in those with normal PTFV1 (both log-rank p < 0.001; Fig. 2B and C). The multivariate analyses adjusted for age, sex, comorbidities, laboratory measurements, and the severity of CAD showed that abnormal PTFV1 was independently associated with cardiac death (adjusted HR 2.20, 95 % CI 1.46–3.30, p < 0.001) and HF hospitalization (adjusted HR 2.39, 95 % CI 1.76–3.24, p < 0.001).

Furthermore, the sensitivity analysis of patients with echocardiographic assessment showed that PTFV1 carried an independent risk for the primary outcome, even after adjustment for LV/LA structural remodeling in the categorical model (adjusted HR 2.13, 95 % CI 1.40–3.24, p < 0.001) and in the continuous model (adjusted HR 1.22, 95 % CI 1.04–1.43, p = 0.016).

Among 265 patients with the primary outcome, 25 (9.4 %) patients experienced new onset of AF during the follow-up period. A sensitivity analysis in 2875 patients free of AF during follow-up showed an independent relationship between abnormal PTFV1 and the primary outcome (adjusted HR 2.53, 95 % CI 1.92–3.32, p < 0.001 in the full-adjusted model).

### 3.4 Subgroup analysis stratified by ACS and CCS

We also investigated the association between P-wave morphology and the primary outcome stratified by the patient demographics, biomarkers and the CAD burden. There were no significant interactions between these variables and abnormal PTFV1 for the primary outcome (Fig. S2). Focusing on CAD subtypes, the multivariate Cox models showed that abnormal PTFV1 was an independent risk for the primary endpoint both in the ACS subgroup (adjusted HR 3.46, 95 % CI 2.11–5.68, p < 0.001) and in the CCS subgroup (adjusted HR 2.02, 95 % CI 1.48–2.76, p < 0.001).

### 3.5 Incremental prognostic value of PTFV1 for the primary outcome

Net reclassification analysis was performed to determine the incremental prognostic value of PTFV1 in predicting the primary outcome, showing that abnormal PTFV1 had the incremental prognostic utility above the comorbidities, biomarkers and the severity of CAD (net reclassification improvement 0.555, 95 % CI 0.430–0.680, p < 0.001).

## 4 Discussion

The main results of the present study are as follows. (i) A total of 18.8 % of patients with CAD had abnormal PTFV1, accompanied by a greater burden of CAD. (ii) PTFV1 was significantly associated with LV and LA morphology in patients with CAD, while the association between LA electrical and structural remodeling was attenuated after multivariable adjustment for potential confounders and LV remodeling. (iii) Abnormal PTFV1 carried a significant risk for adverse cardiovascular outcomes, independent of relevant risk factors, laboratory parameters, and the severity of CAD. This finding remained significant with further adjustment for LV/LA structural remodeling. (iv) The independent relationship between abnormal PTFV1 and a poor prognosis was consistent in the ACS and CCS subgroups. (v) Abnormal PTFV1 had the incremental prognostic utility beyond the conventional risk factors and the CAD burden.

### 4.1 Association between PTFV1 and CAD

In this study, 18.8 % of patients with CAD who underwent PCI had abnormal PTFV1. Previous studies showed the prevalence of abnormal PTFV1 was 7 % in the general population [21,27], 18 % in patients with HF [18], and 29 % in patients with acute myocardial infarction [10]. These results are consistent with our finding of the relatively high frequency of abnormal PTFV1 in the setting of CAD.

PTFV1, which is a prolonged duration and enhanced terminal force amplitude of the P wave, reflects interatrial conduction abnormality and fibrofatty atrial remodeling, which are associated with the amount of LA low-voltage substrate [28]. Several factors may explain the association between CAD and abnormal PTFV1. First, the accumulation of shared risk factors, such as hypertension and CKD, could partially account for this relationship [29,30]. Second, advanced LV fibrotic remodeling in relation to increased PTFV1 can also play a major role in the relationship between abnormal PTFV1 and the CAD burden [8]. Furthermore, atrial ischemia may enhance LA electrical and structural remodeling. In fact, we found that patients with abnormal PTFV1 had worse CAD profiles, including multivessel disease, LMT involvement, and chronic total occlusion. These findings are in line with previous studies, which showed that LA conduction abnormality and structural remodeling were more pronounced with increasing severity of CAD [[31], [32], [33]].

### 4.2 Relationship between P-wave morphological changes and myocardial remodeling

This study showed significant associations between PTFV1 and LV/LA structural remodeling in patients with CAD. Studies using imaging modalities have shown that P-wave indices reflect LV and LA structural remodeling in the settings of AF and HF, as well as CAD [[8], [9], [10], [11],^18^]. However, in the present study, the association between PTFV1 and LA structural remodeling was attenuated after adjusting for potential confounding factors and LV remodeling. Recent imaging studies have demonstrated a positive correlation of P-wave duration with LA size and fibrosis in the general population and patients with AF [9,21], whereas no significant associations were observed for PTFV1 [21,34]. Furthermore, a histological study in patients who received CABG displayed that the extent of atrial fibrosis was greater in the normal PTFV1 group than in the abnormal PTFV1 group [10]. These findings suggest that advanced atrial fibrotic remodeling, characterized by a reduction in the number of electrically active and viable cardiomyocytes, may be associated with prolonged intra-atrial conduction time but is less likely to generate terminal force amplitude, which could explain the attenuated relationship between PTFV1 and LA remodeling.

### 4.3 P-wave morphology and adverse cardiac events in CAD

In the present study, abnormal PTFV1 was independently associated with the adverse long-term outcomes of cardiac death and HF hospitalization after adjustment for age, sex, comorbidities, laboratory parameters, and the severity of CAD. This association was maintained after using PTFV1 as a continuous variable. The prognostic value of abnormal PTFV1 for adverse cardiovascular outcomes has been investigated in some clinical situations [15,18,[35], [36], [37]]. Maheshwari et al. reported that abnormal PTFV1 was an independent risk for sudden cardiac death in 13,580 individuals free of cardiac disease [15]. Baturova et al. also identified abnormal PTFV1 as an independent predictor for HF-related events in 670 patients with HF who were candidates for cardiac resynchronization therapy [18]. In addition, Liu et al. showed that abnormal PTFV1 was independently related to cardiac death or HF hospitalization in 185 patients with acute myocardial infarction [35]. Our findings extend these results to a larger population of comprehensive CAD, namely ACS and CCS. We found that the prognostic value of abnormal PTFV1 was independent of myocardial structural remodeling and remained significant in the subsets of ACS and CCS.

### 4.4 Clinical implications

The independent and robust association between PTFV1 and long-term adverse cardiovascular outcomes could aid in early, easily available, and cost-effective risk stratification after coronary artery revascularization in patients with CAD. Although PTFV1 is a cardiac-specific marker, its prognostic value was also significant for all-cause mortality, suggesting its applicability to daily clinical practice. An incremental prognostic value of abnormal PTFV1 beyond traditional risk factors may help identify high-risk CAD patients when incorporated into currently available risk models or guidelines [[38], [39], [40]], warranting further multicenter and large-scale studies. Recently, emerging evidence has demonstrated the clinical utility of machine learning approaches based on P-wave morphologies for identifying high-risk patients [41]. The high reproducibility of PTFV1 between automatic and manual measurements suggest that PTFV1 may be applicable to machine learning approaches, thereby facilitating accessible and economical risk stratification. Further studies are warranted to investigate whether patients with abnormal PTFV1 would benefit from intensive care, such as strict management of atherosclerotic risk factors and introduction of cardioprotective medications (e.g., sodium glucose co-transporter 2 inhibitors and angiotensin receptor neprilysin inhibitors) [42,43]. Moreover, examining the effect of pharmacological and interventional therapies on PTFV1 in relation to cardiovascular outcomes could enhance the prognostic utility of PTFV1 in patients with CAD.

### 4.5 Limitations

The single-center, retrospective, observational nature of the present study could not exclude potential unmeasured confounders regarding the association between PTFV1 and clinical events. Further multicenter studies are required to confirm the prognostic value of PTFV1 and the applicability to other study population. Although the definition of abnormal PTFV1 was based on the cut-point related to adverse cardiovascular outcomes in previous studies [12,15,17,21], different cut-off value may enhance sensitivity or the prognostic value of PTFV1, which should be investigated in future research. In addition, the lack of other ECG parameters including abnormal Q wave and left bundle branch block may also affect our findings, while previous study demonstrated an independent prognostic value of PTFV1 beyond Q-wave abnormalities in patients with AMI [35]. We could not exclude the potential bias arising from the lack of echocardiography on all patients regarding the association between PTFV1 and cardiovascular events. We also could not conclude the relationship between LA electrical and structural remodeling in patients with CAD due to a lack of LA volumetric evaluation, which needs to be clarified in future studies with LA volume and strain analysis. Furthermore, although the sensitivity analysis suggested that an effect of incident AF on these clinical events may have been limited in our study, we could not completely exclude a potential bias in the incident AF, given the possibility of undiagnosed asymptomatic cases.

## 5 Conclusions

In patients with CAD who undergo PCI, abnormal PTFV1 is a robust and independent risk factor for adverse cardiovascular outcomes. Future studies are required to determine whether intensive lifestyle and pharmacological intervention in patients with CAD and abnormal PTFV1 have a preventive effect on cardiovascular events.

## CRediT authorship contribution statement

**Kazutoshi Hirose:** Writing – original draft, Visualization, Validation, Methodology, Investigation, Formal analysis, Data curation, Conceptualization. **Hiroyuki Kiriyama:** Writing – review & editing, Project administration, Methodology, Investigation. **Shun Minatsuki:** Writing – review & editing, Supervision, Methodology. **Yugo Nagae:** Methodology, Investigation. **Tatsuki Furusawa:** Writing – review & editing. **Takashi Hiruma:** Writing – review & editing. **Atsushi Kobayashi:** Writing – review & editing, Investigation. **Masataka Sato:** Writing – review & editing, Investigation. **Shinnosuke Sawano:** Writing – review & editing, Software, Investigation, Data curation. **Tatsuya Kamon:** Writing – review & editing, Investigation. **Hiroki Shinohara:** Writing – review & editing, Software, Investigation, Data curation. **Akihito Saito:** Writing – review & editing. **Satoshi Kodera:** Writing – review & editing, Software, Resources. **Junichi Ishida:** Writing – review & editing. **Hiroyuki Morita:** Writing – review & editing. **Norihiko Takeda:** Supervision.

## Data availability statement

The data that support the findings of this study are available from the corresponding author upon reasonable request.

## Funding sources

None.

## Declaration of competing interest

The authors declare that they have no known competing financial interests or personal relationships that could have appeared to influence the work reported in this paper.
