# Supplementary material for: Prevalence, associated factors, and prognostic value of P wave abnormality in patients with coronary artery disease
Source: Int J Cardiol Cardiovasc Risk Prev. 2025 Oct 24;27:200533. doi: 10.1016/j.ijcrp.2025.200533 (PMC12641559; doi:10.1016/j.ijcrp.2025.200533)

| Table S1. Baseline characteristics in patients with echocardiographic measurement. | | | |
| --- | --- | --- | --- |
|  | Abnormal PTFV1 (N=296) | Normal PTFV1 (N=1,174) | p value |
| PTFV1, mm∙s | 0.050 (0.045-0.061) | 0.019 (0.012-0.028) | N/A |
| Age, years | 69±11 | 68±11 | 0.401 |
| Men, n (%) | 233 (78.7) | 920 (78.4) | 0.895 |
| Body mass index, kg/m^2^ | 24.3±4.2 | 24.4±3.9 | 0.717 |
| Atherosclerotic risk factors |  |  |  |
| Current smoking, n (%) | 188 (63.5) | 761 (64.8) | 0.674 |
| Hypertension, n (%) | 260 (87.8) | 987 (84.1) | 0.107 |
| Diabetes mellitus, n (%) | 154 (52.0) | 638 (54.3) | 0.475 |
| Dyslipidemia, n (%) | 232 (78.4) | 1,002 (85.4) | 0.004 |
| CKD, n (%) | 134 (45.3) | 456 (38.8) | 0.044 |
| Cardiovascular comorbidities |  |  |  |
| ACS, n (%) | 77 (26.0) | 316 (26.9) | 0.754 |
| Prior myocardial infarction, n (%) | 45 (15.2) | 121 (10.3) | 0.017 |
| History of PCI, n (%) | 42 (14.2) | 173 (14.7) | 0.812 |
| History of CABG, n (%) | 44 (14.9) | 62 (5.3) | <0.001 |
| HF hospitalization, n (%) | 20 (6.8) | 25 (2.1) | <0.001 |
| Stroke/TIA, n (%) | 27 (9.1) | 110 (9.4) | 0.896 |
| Laboratory data |  |  |  |
| BNP, pg/ml (n=1,421) | 101 (38-331) | 42 (18-107) | <0.001 |
| CRP, mg/dl (n=1,466) | 0.16 (0.06-0.45) | 0.10 (0.05-0.29) | <0.001 |
| Echocardiographic parameters |  |  |  |
| Interventricular septal thickness, mm | 10.2±1.9 | 9.9±1.8 | 0.003 |
| Posterior wall thickness, mm | 10.0±1.8 | 9.5±1.7 | <0.001 |
| LV end-diastolic dimension, mm | 49.2±8.2 | 47.5±6.3 | 0.003 |
| LV end-systolic dimension, mm | 34.0±10.3 | 31.3±7.2 | 0.001 |
| LV ejection fraction, % | 58.1±16.1 | 61.9±12.0 | 0.007 |
| LV mass index g/m^2^ | 108.4 (86.9-130.9) | 94.6 (79.3-113.0) | <0.001 |
| LA dimension, mm | 40.6±7.3 | 38.9±6.3 | <0.001 |
| E/A ratio | 0.78 (0.65-1.07) | 0.79 (0.66-0.97) | 0.903 |
| E/e’ ratio | 11.5 (9.0-15.9) | 10.0 (8.0-12.6) | <0.001 |
| Angiographic findings |  |  |  |
| LMT lesion, n (%) | 44 (14.9) | 96 (8.2) | <0.001 |
| Multivessel disease, n (%) | 227 (76.7) | 870 (74.1) | 0.361 |
| Chronic total occlusion, n (%) | 80 (27.0) | 220 (18.7) | 0.002 |
| A = late diastolic transmitral flow velocity, ACS = acute coronary syndrome, BNP = brain natriuretic peptide, CABG = coronary artery bypass grafting, CKD = chronic kidney disease, CRP = C-reactive protein, E = early diastolic transmitral flow velocity, e’ = early diastolic mitral annular velocity, eGFR = estimated glomerular filtration rate, HDL = high density lipoprotein, HF = heart failure, LA = left atrium, LDL = low density lipoprotein, LMT = left main trunk artery, LV = left ventricle, PCI = percutaneous coronary intervention, PTFV1 = P-wave terminal force in V1, RAAS = renin-angiotensin-aldosterone system and TIA = transient ischemic attack. | | | |

| Table S2. Factors associated with PTFV1 (per 1-SD increase). | | | | |
| --- | --- | --- | --- | --- |
|  | Univariable | | Multivariable | |
|  | S(β) (95% CI) | p value | S(β) (95% CI) | p value |
| Age, years | 0.072 (0.002 to 0.012) | 0.005 | 0.001 (-0.005 to 0.005) | 0.961 |
| Men | 0.021 (-0.038 to 0.088) | 0.431 |  |  |
| Body mass index, kg/m^2^ | -0.011 (-0.016 to 0.010) | 0.687 |  |  |
| Current smoking | -0.023 (-0.079 to 0.029) | 0.371 |  |  |
| Hypertension | 0.069 (0.025 to 0.170) | 0.008 | 0.026 (-0.034 to 0.108) | 0.312 |
| Diabetes mellitus | 0.013 (-0.039 to 0.066) | 0.611 |  |  |
| Dyslipidemia | -0.077 (-0.177 to -0.036) | 0.003 | -0.026 (-0.107 to 0.034) | 0.310 |
| CKD | 0.091 (0.042 to 0.147) | <0.001 | -0.042 (-0.098 to 0.012) | 0.122 |
| ACS | -0.032 (-0.095 to 0.022) | 0.221 |  |  |
| Prior myocardial infarction | 0.059 (0.012 to 0.176) | 0.024 | 0.004 (-0.074 to 0.086) | 0.885 |
| History of PCI | -0.012 (-0.091 to 0.056) | 0.634 |  |  |
| History of CABG | 0.189 (0.272 to 0.469) | <0.001 | 0.110 (0.111 to 0.317) | <0.001 |
| HF hospitalization | 0.143 (0.271 to 0.569) | <0.001 | 0.045 (-0.016 to 0.278) | 0.108 |
| Stroke/TIA | 0.004 (-0.082 to 0.097) | 0.865 |  |  |
| Log BNP | 0.323 (0.464 to 0.631) | <0.001 | 0.190 (0.214 to 0.432) | <0.001 |
| Log CRP | 0.127 (0.117 to 0.273) | <0.001 | 0.018 (-0.051 to 0.106) | 0.495 |
| LV ejection fraction, % | -0.172 (-0.017 to -0.009) | <0.001 | 0.001 (-0.004 to 0.005) | 0.960 |
| LV mass index, g/m^2^ | 0.245 (0.007 to 0.010) | <0.001 | 0.090 (0.001 to 0.005) | 0.002 |
| LA dimension, mm | 0.169 (0.018 to 0.034) | <0.001 | 0.026 (-0.004 to 0.012) | 0.355 |
| E/e’ ratio | 0.275 (0.043 to 0.062) | <0.001 | 0.135 (0.015 to 0.036) | <0.001 |
| LMT lesion | 0.107 (0.097 to 0.273) | <0.001 | 0.038 (-0.021 to 0.151) | 0.136 |
| Multivessel disease | 0.028 (-0.027 to 0.093) | 0.276 |  |  |
| Chronic total occlusion | 0.102 (0.064 to 0.193) | <0.001 | 0.017 (-0.044 to 0.086) | 0.527 |
| ACS = acute coronary syndrome, BNP = brain natriuretic peptide, CABG = coronary artery bypass grafting, CI = confidence interval, CKD = chronic kidney disease, CRP = C-reactive protein, E = early diastolic transmitral flow velocity, e’ = early diastolic mitral annular velocity, HF = heart failure, LA = left atrium, LMT = left main trunk artery, LV = left ventricle, PCI = percutaneous coronary intervention, PTFV1 = P-wave terminal force in V1, SD = standard deviation and TIA = transient ischemic attack. | | | | |

| Table S3. Cox regression models in the association of PTFV1 with the composite outcome of all-cause mortality and HF hospitalization. | | | | |
| --- | --- | --- | --- | --- |
|  | Categorical model  (PTFV1 >0.04 mm∙s) | | Continuous model  (per 1-SD increase) | |
|  | HR (95% CI) | p value | HR (95% CI) | p value |
| Univariate model | 2.19 (1.81-2.64) | <0.001 | 1.33 (1.24-1.41) | <0.001 |
| Multivariate model | 1.52 (1.24-1.86) | <0.001 | 1.12 (1.04-1.21) | 0.005 |
| Multivariate model = adjusted for age, sex, body mass index, current smoking, hypertension, diabetes mellitus, dyslipidemia, CKD, ACS, prior myocardial infarction, history of PCI, CABG and HF hospitalization, stroke/transient ischemic attack, log BNP, log CRP, LMT lesion, multivessel disease and chronic total occlusion. ACS = acute coronary syndrome, BNP = brain natriuretic peptide, CABG = coronary artery bypass grafting, CI = confidence interval, CKD = chronic kidney disease, CRP = C-reactive protein, HF = heart failure, HR = hazard ratio, LMT = left main trunk artery, PCI = percutaneous coronary intervention, PTFV1 = P-wave terminal force in V1 and SD = standard deviation. | | | | |

**Figure S1:** Kaplan–Meier survival curves of the composite outcome of all-cause death and HF hospitalization stratified by the PTFV1 status. HF = heart failure and PTFV1 = P-wave terminal force in V1.


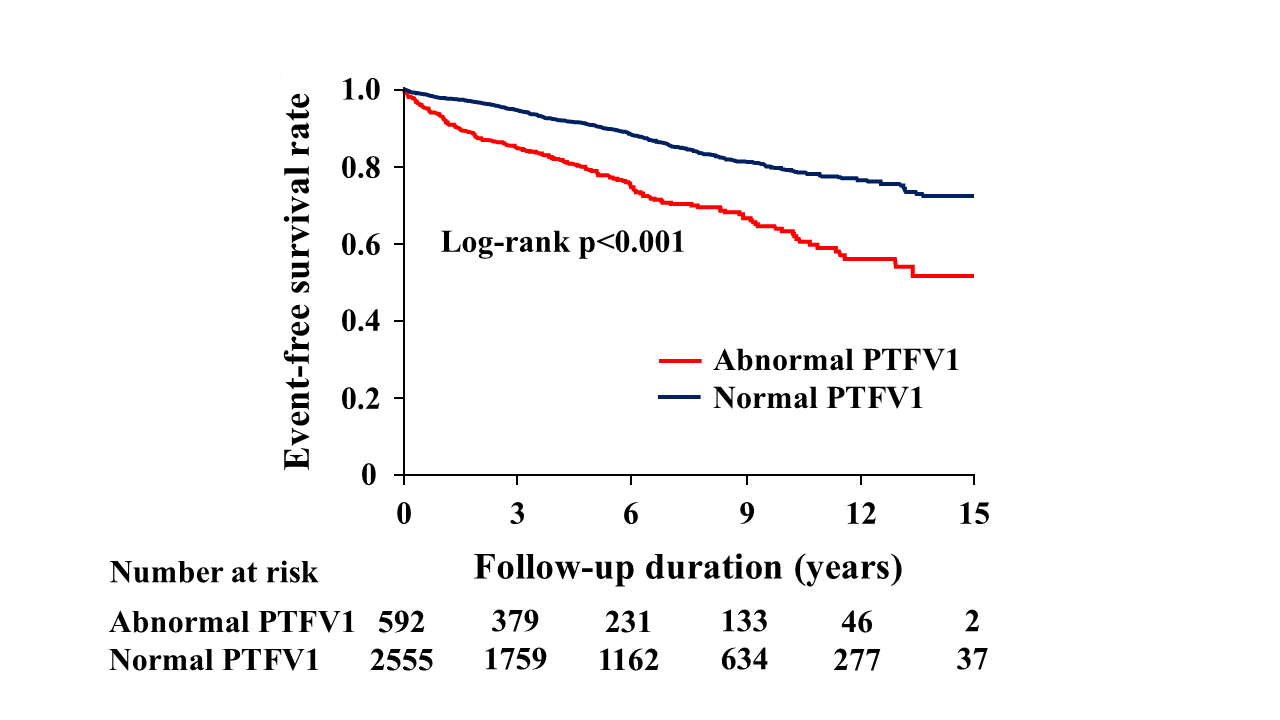


**Figure S2:** Subgroup analyses of the primary outcome. ACS = acute coronary syndrome, BNP = brain natriuretic peptide, CABG = coronary artery bypass grafting, CAD = coronary artery disease, CCS = chronic coronary syndrome, CI = confidence interval, CKD = chronic kidney disease, CRP = C-reactive protein, HF = heart failure, HR = hazard ratio, LMT = left main trunk artery, MI = myocardial infarction, PCI = percutaneous coronary intervention, PTFV1 = P-wave terminal force in V1 and TIA = transient ischemic attack.


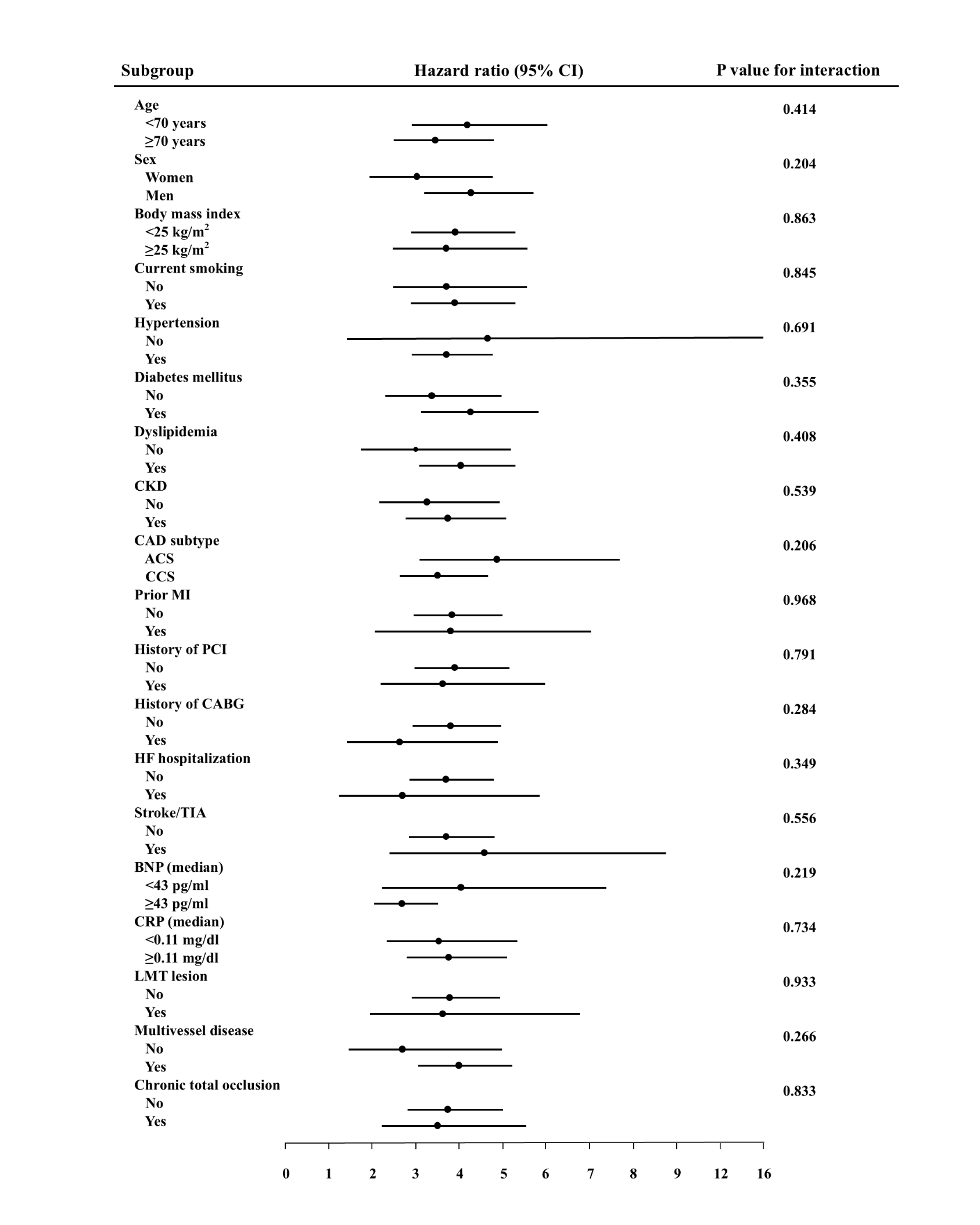

Supplement: Multimedia component 1 [file mmc1.docx]
